# Secuelas clínicas y neurodesarrollo de pacientes pediátricos con toxoplasmosis congénita en un centro de cuarto nivel

**DOI:** 10.7705/biomedica.7206

**Published:** 2024-11-06

**Authors:** Sebastián Posada-Bustos, Ana Cristina Mariño, Eugenia Espinosa-García

**Affiliations:** 1 Servicio de Neurología Pediátrica, Hospital Militar Central, Facultad de Medicina, Universidad Militar Nueva Granada, Bogotá, D. C., Colombia Universidad Militar Nueva Granada Facultad de Medicina Universidad Militar Nueva Granada Bogotá D. C Colombia; 2 Servicio de Infectología Pediátrica, Hospital Militar Central, Facultad de Medicina, Universidad Militar Nueva Granada, Bogotá, D. C., Colombia Universidad Militar Nueva Granada Facultad de Medicina Universidad Militar Nueva Granada Bogotá D. C Colombia

**Keywords:** toxoplasma, toxoplasmosis, toxoplasmosis congénita, coriorretinitis, trastornos del neurodesarrollo, discapacidad intelectual, Colombia, Toxoplasma, toxoplasmosis, congenital, toxoplasmosis, chorioretinitis, neurodevelopmental disorders, intellectual disability, Colombia

## Abstract

**Introducción.:**

La toxoplasmosis congénita es una enfermedad parasitaria de importante prevalencia a nivel mundial, con gran morbilidad y afectación del neurodesarrollo en pacientes pediátricos.

**Objetivo.:**

Describir las secuelas y valorar el neurodesarrollo de pacientes pediátricos con toxoplasmosis congénita en el Hospital Militar Central del 2013 al 2020.

**Materiales y métodos.:**

Se trata de un estudio observacional, descriptivo y de corte transversal, con componente analítico, que incluyó los pacientes pediátricos con diagnóstico de toxoplasmosis congénita que consultaron al Hospital Militar Central durante el periodo de enero de 2013 a diciembre de 2020. En los niños menores de seis años, se utilizó la escala de neurodesarrollo *Ages and Stages Questionnaires 3.*

**Resultados.:**

Se incluyeron 45 pacientes con toxoplasmosis congénita confirmada, con una media de edad de 5,9 años; 60 % eran de sexo masculino. El 11,2 % estaban sintomáticos al nacer y el 33 % presentó coriorretinitis. Durante el seguimiento, el 73 % presentó secuelas oftalmológicas; el 64 %, tenía calcificaciones en la tomografía computarizada; el 4,4 %, hidrocefalia; el 11,2 %, parálisis cerebral, y el 13,4 %, epilepsia focal. El 58 % de los menores de seis años presentó compromiso del neurodesarrollo y el 62 % de los mayores de seis años tenía déficit cognitivo. En esta cohorte, el 68 % de los pacientes recibió tratamiento posnatal. Se obtuvo una asociación estadísticamente significativa entre no recibir tratamiento y las secuelas oftalmológicas (OR = 5,2; p < 0,001).

**Conclusiones.:**

La toxoplasmosis congénita se asoció con secuelas a largo plazo, similares a las descritas en otras series de casos latinoamericanos. Es de suma importancia hacer un diagnóstico temprano, con evaluación, tratamiento y seguimiento interdisciplinario oportunos en los pacientes colombianos para mejorar su pronóstico.

## Abstract (en)

**Introduction.:**

Congenital toxoplasmosis is a highly prevalent parasitic disease worldwide, with a high burden of disease and neurodevelopmental involvement in pediatric patients.

**Objective.:**

To describe the clinical sequelae and neurodevelopmental state of pediatric patients with congenital toxoplasmosis at the *Hospital Militar Central* during 2013 to 2020.

**Materials and methods.:**

We conducted an observational, descriptive, cross-sectional study with an analytical component, including pediatric patients diagnosed with congenital toxoplasmosis. Patients consulted the *Hospital Militar Central* from January 2013 to December 2020. The Ages and Stages Questionnaires 3 neurodevelopmental scale was applied to children under six years old.

**Results.:**

Forty-five patients with confirmed congenital toxoplasmosis were included, with a mean age of 5.9 years; 60% were male; 11.2 % were symptomatic at birth, and 33% presented chorioretinitis. During the follow-up, 73% presented ophthalmologic sequelae, 64% cerebral calcifications, 4.4% hydrocephalus, 11.2% cerebral palsy, and 13.4% focal epilepsy. In children under six years old, 58% presented neurodevelopmental compromise, and in those over six years old, 62% had cognitive deficits. In this cohort, 68% of the patients received posnatal treatment, with a statistically significant association between not receiving treatment and ophthalmological sequelae (OR = 5.2; p < 0.001).

**Conclusions.:**

Congenital toxoplasmosis is associated with important long-term sequelae similar to those described in several Latin American series. These findings highlight the importance of early diagnosis, evaluation, treatment, and timely interdisciplinary follow-up of patients in our country to improve their prognosis.

La toxoplasmosis es una enfermedad parasitaria de importante prevalencia a nivel mundial: se estima que más de un tercio de la población se encuentra afectada ^1^. Por lo general, la infección aguda en mujeres gestantes es asintomática y la principal forma de detectarla es mediante pruebas serológicas. La tasa de prevalencia identificada es de 3 a 10 por cada 1.000 mujeres embarazadas ^2^. La transmisión del parásito de la madre al hijo aumenta gradualmente con el progreso de la gestación, es decir, a mayor edad gestacional, es mayor la probabilidad de infección fetal y, a menor edad gestacional, hay menor riesgo de infección fetal ^3^. En Europa, la seroprevalencia en mujeres embarazadas es muy variable (12 a 54 %), mientras que, en Estados Unidos, es del 9 % y ha disminuido en los últimos años ^3^^,^^4^.

En Colombia, de acuerdo con el Estudio Nacional de Salud realizado entre 1977 y 1980, la tasa de seroprevalencia observada en mujeres en edad fértil varió entre el 42,5 y el 54,4 % ^5^. En una revisión del 2021, se demostró la presencia de esta infección en todas las zonas ecológicas de Colombia, desde las tierras secas y bajas hasta los bosques húmedos montañosos, con predominio del 82 % en las zonas urbanas ^6^. La tasa de infecciones adquiridas en los recién nacidos durante el embarazo en Colombia varía entre el 1,3 y el 8,4 % ^5^.

La prevalencia mundial de la toxoplasmosis congénita en recién nacidos varía de 1 a 10 por 10.000 nacidos vivos ^5^. En Colombia, se estima entre 2 y 10 por cada 1.000 nacidos vivos, para un total de 600 a 3.000 niños nacidos por año con infección congénita ^7^.

El riesgo de secuelas en el feto es inversamente proporcional a la edad gestacional, es decir, a menor edad gestacional, hay mayor riesgo de secuelas y lesiones, mientras que, a mayor edad gestacional, el riesgo de secuelas es menor ^7^. La enfermedad en el hijo se manifiesta en la vida intrauterina y extrauterina. El compromiso de quienes presentan la infección varía según el grado de la lesión: desde presentación grave y generalizada, con exantema maculopapular, púrpura, neumonía, ictericia prolongada y hepatoesplenomegalia, hasta alteraciones del sistema nervioso central dadas por hidrocefalia, calcificaciones intracerebrales, epilepsia, microcefalia o microftalmia con coriorretinitis o *sinella,* o infección subclínica ^2^. A largo plazo, estos pacientes pueden presentar retraso del neurodesarrollo o déficit cognitivo, alteraciones motoras, hipoacusia y alteraciones visuales ^2^. El riesgo de secuelas es mayor (hasta el 92 %) en pacientes no tratados en la época gestacional o posnatal ^2^.

No se cuenta con estudios de seguimiento de secuelas y alteraciones neurológicas a largo plazo en pacientes con toxoplasmosis congénita en Colombia, ni se conocen la oportunidad y el impacto del tratamiento prenatal y el posnatal en esos resultados a largo plazo.

En este contexto, este estudio tuvo como objetivo describir las secuelas clínicas y el estado del neurodesarrollo en pacientes pediátricos con toxoplasmosis congénita durante el periodo 2013-2020 en el Hospital Militar Central, hospital de referencia del sistema de sanidad militar colombiano para esta enfermedad.

## Materiales y métodos

### Diseño del estudio y población

Se llevó a cabo un estudio de corte transversal, descriptivo, observacional, con componente analítico, mediante el análisis de los datos obtenidos de las historias clínicas de los pacientes pediátricos (menores de 18 años) con diagnóstico de toxoplasmosis congénita, confirmado según los criterios de la guía colombiana de diagnóstico, desde enero del 2013 hasta diciembre del 2020.

Los pacientes habían sido remitidos a consulta de neurología e infectología pediátrica del Hospital Militar Central ^7^. Se excluyeron pacientes con otras infecciones congénitas, como citomegalovirus, sífilis, rubeola, virus del Zika, herpes o fiebre de chikunguña, y aquellos sin valoración por oftalmología o estudios de imágenes diagnósticas.

### Medidas y variables

Se consideraron las variables sociodemográficas y clínicas, y los antecedentes perinatales que pudieran condicionar un gran riesgo neurológico adicional (consumo de tóxicos o sustancias psicoactivas durante el embarazo, enfermedades maternas o fetales, malformaciones, asfixia o enfermedades perinatales). Se registró el trimestre de adquisición de la infección gestacional por toxoplasma, el tratamiento recibido y su duración. Posteriormente, se tomaron las variables: edad de diagnóstico de la toxoplasmosis congénita, tratamiento posnatal y duración, compromiso clínico congénito y secuelas a largo plazo. En la última valoración registrada en el seguimiento por el servicio de neurología pediátrica, se consideraron las secuelas clínicas (epilepsia, hidrocefalia, microcefalia, hipoacusia), el compromiso ocular según la valoración de oftalmología, y los estudios de audición y de neuroimágenes.

Se tomaron los resultados de la escala *Ages and Stages Questionnaires* (ASQ 3). Con este instrumento se evalúa cada una de las áreas del neurodesarrollo (motora, lingüística, social y cognitiva) de los pacientes entre los seis meses y los seis años ^8^. La escala se encuentra disponible en español y está validada en más de 18.000 niños, desde su aplicación inicial en Estados Unidos y, posteriormente, en países de habla hispana, como España, Chile, Argentina, México y Brasil, con excelentes propiedades psicométricas: sensibilidad del 87,4 % y especificidad del 95,7 % ^9^^,^^10^.

Para los pacientes mayores de seis años, se tomaron los valores del coeficiente intelectual medidos mediante pruebas neuropsicológicas, indicadas previamente por sospecha de déficit cognitivo.

### Análisis estadístico

Se utilizaron medidas estadísticas descriptivas para caracterizar la población. Se seleccionaron medidas de tendencia central para las variables no continuas y se elaboraron tablas de distribución de proporciones para las variables discontinuas. Se estimó la asociación entre la evaluación general del neurodesarrollo y sus áreas (personal-social, lingüística, cognitiva y motora), y la variable de recibir o no recibir tratamiento para la toxoplasmosis congénita posnatal. Para esto, se utilizó la prueba de ji al cuadrado (c^2^) para las variables discontinuas y se calculó la razón de momios *(odds ratio,* OR) para las variables binarias, estratificadas por trimestre de infección. La evaluación estratificada se realizó mediante la prueba estadística de Mantel-Haenszel para establecer la homogeneidad entre los estratos.

En cuanto a las variables continuas, se usó la prueba de DAgostino para determinar si tenían distribución normal y, luego, la prueba U de MannWhitney para establecer diferencias estadísticamente significativas entre los dos grupos. Se tomó un valor de p < 0,05 como estadísticamente significativo para todos los casos. Para el análisis de los datos, se usó el *software* Real Statistics, versión 7.9, de septiembre de 2021.

### Aspectos éticos

Este estudio fue aprobado por el Comité de Investigación del Hospital Militar Central, como investigación sin riesgo de acuerdo con la Resolución 8430 de 1993 del Ministerio de Salud de Colombia.

## Resultados

Entre el 2013 y el 2020, 45 pacientes con diagnóstico confirmado de toxoplasmosis congénita asistieron a control por neurología e infectología pediátrica al Hospital Militar Central. La media de la edad fue de 5,9 años, el 53,3 % correspondió a menores de seis años y el 60 % eran de sexo masculino. En relación con los factores de riesgo gestacionales, tres mujeres habían padecido enfermedades durante el embarazo (diabetes, preeclampsia e hipotiroidismo), controladas en su momento. El acceso al control prenatal fue adecuado, al menos, con seis controles en todos los casos.

**Figura 1:**
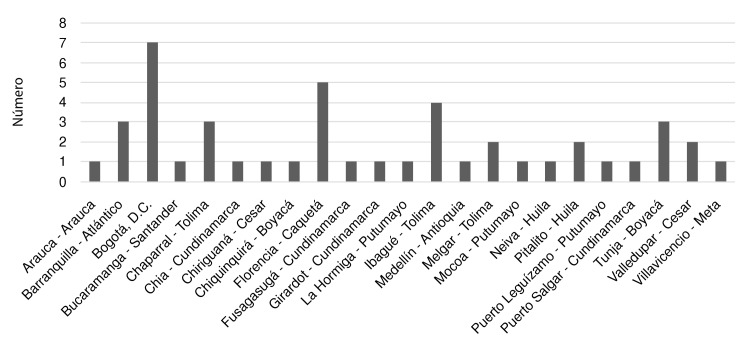
Procedencia de pacientes con toxoplasmosis congénita

Se obtuvo una muestra de diferentes lugares de procedencia del país, con representación de las regiones Caribe, Andina, Orinoquía y Amazonía, así como de Bogotá. Tolima fue el departamento con más casos (n = 9; 20 %), seguido de la ciudad de Bogotá (n = 7; 15,5 %) y Boyacá (n = 4; 8,9 %) (figura 1).

El 60 % de las madres de los 45 pacientes presentaron la infección por toxoplasma en el tercer trimestre de gestación. El 53,3 % recibió tratamiento para toxoplasmosis gestacional -con espiramicina en todos los casos- desde la seroconversión hasta el momento del parto (5,2 semanas en promedio). La mayoría de los pacientes (n = 39; 86,7 %) nacieron a término, mientras que seis (13,3 %) fueron prematuros: un paciente nació en la semana 34, dos en la semana 35 y tres en la semana 36. En todos los casos, la adaptación neonatal fue adecuada, sin datos de asfixia perinatal y un Apgar mayor de 7 a los cinco minutos. Los datos sociodemográficos de los pacientes se muestran en el cuadro 1.

**Cuadro 1:** Datos sociodemográficos (n = 45)

Variable		Medidas de dispersión	
Edad			
	Media (años, DE)	5,9	3,08
	Mediana (años, DE)	5,3	
	Menores de seis años (n, %)	24	53,3
	Mayores de seis años (n, %)	21	46,7
Sexo (n, %)			
	Femenino	18	40
	Masculino	27	60
Número de embarazos (media, DE)		1,7	0,9
Edad materna (años) (media, DE)		25,6	5,54
Trimestre de infección (n, %)			
	Primero	6	13,3
	Segundo	12	26,7
	Tercero	27	60
Tratamiento gestacional (n, %)		24	53,3
Tiempo de tratamiento (semanas) (media, DE)		5,2	7,5
Vía de nacimiento			
	Vaginal (n, %)	32	71,1
	Cesárea (n, %)	13	28,9
Semanas de embarazo (media)		38,2	
	Prematuros (n, %)	6	13,3
Peso al nacer (g) (media, DE)		3.034	500,5
Talla al nacer (cm) (media, DE)		49,1	3,2
Edad de diagnóstico (meses)		9,4	15,2
	1-12 (n, %)	33	73,3
	13-48 (n, %)	12	26,7
Tratamiento congénito (n, %)		31	68,9
Duración del tratamiento (meses) (media, DE)		12	2,3

DE: desviación estándar

Durante el periodo neonatal, en 7 (15,5 %) de los pacientes se presentaron enfermedades, como taquipnea transitoria en tres, ictericia neonatal manejada con fototerapia en dos, conducto arterioso persistente con repercusión hemodinámica en uno y enfermedad de membrana hialina en otro. Los neonatos con manifestaciones clínicas asociadas con toxoplasmosis congénita, fueron 5 (11,1 %). En el examen oftalmológico practicado de forma temprana por sospecha de toxoplasmosis, se encontró compromiso por coriorretinitis en 15 (33,3 %) de los niños. La estancia hospitalaria promedio fue de 3,2 días para todos los pacientes sintomáticos de la infección al nacer.

La edad promedio al momento del diagnóstico de toxoplasmosis congénita fue de 9,4 meses. El diagnóstico se hizo antes del primer año en 33 (73,3 %), en el primer mes en 21 (46,7 %), y después del primer año y hasta los 48 meses, en 12 (26,7 %).

De los 45 pacientes, 31 (68,9 %) recibieron tratamiento desde el momento del diagnóstico posnatal, cuya media de duración fue de 12 meses. En estos niños, el esquema de tratamiento consistió en sulfadoxina o sulfadiazina y pirimetamina en 6, sulfadiazina, pirimetamina y ácido folínico en 12, y sulfadoxina, pirimetamina y ácido folínico en 13 pacientes. En dos casos con diagnóstico prenatal, por hiperproteinorraquia y coriorretinitis activa, se administraron corticoides al nacer.

Del total de pacientes, 33 (73,3 %) tenían cicatrices coriorretinianas. En el examen audiológico, solo 3 (6,7 %) presentaron hipoacusia, la cual fue moderada y bilateral en dos, y grave y bilateral en el otro.

A lo largo del seguimiento, se practicó ecografía transfontanelar en 21 (46,7 %) de los casos, con resultados anormales en 3 (6,7 %) por presencia de calcificaciones o hidrocefalia; en el resto de los pacientes (n = 18), la ecografía fue normal. La tomografía computarizada de cráneo fue el examen de elección en todos los casos y mostró hallazgos anormales en 31 (68,9 %), entre estos, se encontraron calcificaciones cerebrales en 20 (64,5 %). En los nueve pacientes con mayor compromiso clínico, se practicó resonancia magnética de cerebro; del total de 45 casos, este estudio mostró dilatación del sistema ventricular en 7 (15,6 %), hidrocefalia derivada en 2 (4,4 %) y encefalomalacia asociada en cuatro (8,9 %). En los seis pacientes con epilepsia focal, se hallaron calcificaciones cerebrales.

Las secuelas de la infección se establecieron durante el seguimiento de los pacientes. En 33 (73,3 %) de ellos, se presentó compromiso de la agudeza visual relacionado con las cicatrices coriorretinianas y un paciente presentó reactivación de la coriorretinitis. Durante el periodo de cinco años de seguimiento, un paciente falleció debido a complicaciones de una infección respiratoria grave.

En los 15 pacientes menores de seis años, se evaluó el neurodesarrollo mediante la escala *Ages and Stages Questionnaires* (ASQ 3); en los restantes, los datos se tomaron de las consultas de control de neurología pediátrica. Se encontró que 14 (58,5 %) de los menores de seis años presentaba algún tipo de alteración del neurodesarrollo. Además, el 13 (62 %) de los mayores de seis años presentaban déficit cognitivo. Entre estos últimos y según la valoración neurosicológica, el déficit cognitivo fue: leve, con un promedio del coeficiente intelectual de 62, en 8 (38,1 %); moderado, con un promedio de 46,3, en 3 (14,2 %); y grave, sin datos sobre el coeficiente, en 2 (9,5 %); además, en 1 (4,8 %), se evidenció trastorno de las habilidades escolares y, en cognitivas (cuadro 2).

**Cuadro 2:** Síntomas y secuelas clínicas en pacientes con toxoplasmosis congénita (n = 45)

Síntomas o secuela		Pacientes	Porcent
		(n)	(%)
Síntomas y signos por toxoplasmosis al nacer		5	11,1
	- Trombocitopenia e ictericia	3	
	- Hidrocefalia	1	
	- Microcefalia	1	
Coriorretinitis al nacer		15	33,3
	Coriorretinitis (diagnóstico posnatal)	33	73,3
	- Unilateral	28	
	- Bilateral	5	
Hipoacusia		3	6,7
	- Moderada bilateral	2	
	- Grave bilateral	1	
Imagen diagnóstica			
	Tomografía computarizada de cráneo	45	100,0
	- Normal	14	31,1
	- Calcificaciones bihemisféricas	23	51,1
	- Calcificaciones unilaterales	3	6,7
	-Ventriculomegalia y calcificaciones bilaterales	3	6,7
	- Hidrocefalia y encefalomalacia	2	4,4
Secuelas oftalmológicas		33	73,3
	- Ambliopía unilateral	24	53,3
	- Ambliopía bilateral	6	13,3
	- Ceguera unilateral	3	6,7
	- Sin déficit visual	12	26,7
Secuelas neurológicas			
	Parálisis cerebral espástica	5	11,1
	-GMFCSI	2	
	- GMFCS III	2	
	- GMFCS V	1	
Hidrocefalia		2	4,4
Epilepsia focal estructural		6	13,3
Neurodesarrollo en menores de 6 años			
	- Compromiso del neurodesarrollo	14	58,5
	A. Retraso global	3	12,5
	B. Retraso de lenguaje	6	25
	C. Retraso cognitivo	1	4,2
	D. Retraso motor	1	4,2
	E. Retraso social	1	4,2
	F. Riesgo de retraso lenguaje	1	4,2
Neurodesarrollo en mayores de 6 años			
	- Compromiso del aprendizaje	14	66,7
	A. Déficit cognitivo leve	8	38,1
	B. Déficit cognitivo moderado	3	14,3
	C. Déficit cognitivo grave	2	9,5
	D. Trastorno de habilidades escolares	1	4,8

GMFCS: *Gross Motor Function Classification System*

Aunque las secuelas oftalmológicas se redujeron con el tratamiento prenatal para toxoplasmosis gestacional (OR = 0,78; IC_95%_: 0,2 - 2,97; p = 0,37), al comparar entre los que fueron tratados y los que no lo fueron, no hubo significancia estadística; como tampoco, al comparar las secuelas en el neurodesarrollo o las habilidades cognitivas (OR = 0,78; IC_95%_: 0,2 - 2,97; p = 0,37) ajustadas por trimestre de infección materna, dado que el trimestre de infección se ha demostrado como factor de confusión en varios estudios para evaluar los resultados de la efectividad del tratamiento ^11^ (cuadro 3).

**Cuadro 3:** Evaluación del tratamiento prenatal y riesgo de secuelas clínicas

Variable	OR	IC_95%_	p
Alteración del neurodesarrollo o habilidades cognitivas	Crudo: 0,81	0,22 - 2,79	0,36
	Ajustado*: 0,78	0,20 - 2,97	0,37
Secuelas oftalmológicas	Crudo: 0,64	0,14 - 2,67	0,27
	Ajustado*: 0,65	0,10 - 2,76	0,37

*Ajustado por trimestre de infección

Al comparar los pacientes tratados posnatalmente y los no tratados (ajustados por trimestre de infección), se encontró asociación entre el tratamiento y el déficit cognitivo, sin diferencias estadísticamente significativas (OR = 0,98; IC_95%_: 0,21 - 4,5; p = 0,16). Además, se encontró una asociación estadísticamente significativa entre secuelas oftalmológicas y no haber recibido tratamiento posnatal (OR = 5,2; IC_95%_: 2,6 - 7,82; p = 0,001), ajustado por trimestre de infección materna. No se estableció una asociación estadísticamente significativa con respecto a otras secuelas, como epilepsia o alteraciones en las neuroimágenes (cuadro 4).

**Cuadro 4:** Asociación entre no haber recibido tratamiento posnatal para toxoplasmosis congénita y secuelas clínicas

Variable	OR	IC_95%_		X^2^
Alteración del neurodesarrollo o habilidades cognitivas	Crudo: 0,84	0,22	- 3,3	0,49
	Ajustado: 0,98	0,21	- 4,5	0,16
Secuelas oftalmológicas (ambliopía o ceguera)	Crudo: 4,8	2,6 -	7,85	0.001
	Ajustado*: 5,2	2.6 -	7,82	0.001
Epilepsia	Crudo: 3,6	0,76	- 18,3	0,12
	Ajustado*: 5,93	0,59	- 16,5	0,16
Anormalidad en imágenes diagnósticas	Crudo: 0,63	0,17	- 2,36	0,35
	Ajustado*: 0,66	0,13	- 2,30	0,24

*Ajustado por trimestre de infección

Se corrió el análisis para variables continuas aplicando la prueba de U de Mann-Whitney para identificar diferencias entre los grupos etarios de los pacientes tratados y no tratados después del nacimiento. El análisis estimó diferencias estadísticamente significativas en la edad de evaluación clínica (7,9 años en el grupo que recibió tratamiento versus 5,1 años en el grupo que no recibió; p = 0,003) y en la edad de diagnóstico posnatal (28,9 meses en el grupo que no fue tratado y 6 meses en el grupo tratado; p < 0,001).

Al analizar las variables continuas, se encontró una diferencia estadísticamente significativa en la edad al momento de la evaluación clínica, entre el grupo con tratamiento postnatal (7,9 años) y el grupo que no lo recibió (5,1 años) (p = 0,003). Además, también hubo una diferencia estadísticamente significativa en la edad al momento del diagnóstico postnatal, entre el grupo no tratado (28,9 meses) y el tratado (6 meses) (p=<0,001).

No hubo diferencias estadísticamente significativas en la edad materna, el número del embarazo, las semanas de gestación, ni en el peso o la talla al nacer.

## Discusión

Entre el 2013 y el 2020, 45 pacientes con toxoplasmosis congénita confirmada asistieron a control por neurología e infectología pediátrica. En los estudios previos de Colombia, se han reportado cohortes de 21, 26 y 99 pacientes ^11^^-^^13^.

Se resalta la circulación continua de la infección en el país, con una prevalencia reportada de 1 por cada 1.000 nacidos vivos ^7^. En otros países de Latinoamérica, como Brasil, la prevalencia es similar con una tasa de 1 por cada 1.613 nacidos vivos (6/10.000) ^14^.

La edad media de los pacientes fue de seis años, aproximadamente, con un predominio leve de pacientes de sexo masculino, que correspondieron al 60 %. En los estudios en América y Europa, no se ha reportado predisposición por sexo para la infección congénita ^2^.

En cuanto a la edad de seguimiento, en Brasil y Estados Unidos, la mayoría de pacientes eran menores de seis años ^3^^,^^15^, mientras que en Colombia, solo se habían reportado hasta el año de vida ^11^^,^^12^

Se obtuvo una muestra de procedencia heterogénea, de varias regiones del país. El Hospital Militar Central es un centro de remisión para todo el país y la mayoría de sus pacientes provienen de la región Andina (Bogotá, Tolima, Boyacá), seguida de la Amazonía y el Caribe. En un estudio colombiano sobre tamizaje para toxoplasmosis gestacional, se encontró una gran prevalencia en ciudades como Armenia y Florencia, y una prevalencia intermedia en Bogotá, Barranquilla y Bucaramanga. Estos hallazgos fueron similares a los de reportes previos y, al parecer, están relacionados con el promedio de precipitación, pero no con la temperatura ni la altura sobre el nivel del mar ^16^.

La media de la edad materna al momento del embarazo fue de 25 años. La mayoría de las mujeres gestantes presentó la infección en su segundo embarazo (media de 1,7), lo que concuerda con otros reportes, en los cuales se muestra una relación directamente proporcional entre el riesgo de infección por toxoplasma gestacional y el número de gestaciones ^17^.

Con respecto a la edad gestacional, la toxoplasmosis fue más frecuente en el tercer trimestre (60 %), seguido del segundo (26 %) y el primero (13 %). Estos hallazgos ejemplifican lo descrito en las series, ya que el riesgo de adquirirla aumenta gradualmente con el progreso de la gestación y, por ello, es más común en el último trimestre, aunque su gravedad es menor que en los primeros trimestres ^3^.

En la muestra obtenida, cerca de la mitad (53,3 %) de las madres de los pacientes recibieron tratamiento para toxoplasmosis gestacional hasta el final del embarazo, con espiramicina en todos los casos. Se desconocen los factores asociados con iniciar el tratamiento o no hacerlo, así como con el tiempo de su inicio después de la seroconversión. Aunque este hecho se ha relacionado con mejores resultados a largo plazo, este aspecto no formó parte de los objetivos del presente estudio ^5^^,^^13^.

En el estudio colombiano multicéntrico sobre toxoplasmosis gestacional, la tasa de tratamiento fue del 57 %, similar a la encontrada en el presente estudio (16); en otros países de la región, como Brasil, se reporta una tasa mayor de tratamiento (88 %) ^14^.

Se evidencia la necesidad de mayor observancia de las guías de manejo de la toxoplasmosis en el país, ya que las madres de los pacientes tuvieron un control prenatal apropiado, pero no se logró una adecuada tasa de tratamiento. En un estudio reciente de toxoplasmosis gestacional, la tasa de tratamiento con espiramicina fue del 86 %, lo que impactó en menores tasas de malformaciones cerebrales y alteraciones oftalmológicas ^18^^-^^20^. En un centro del Quindío, se observó que, al implementar las guías de manejo, se mejoró la tasa de diagnóstico y el tratamiento oportuno, con resultados perinatales favorables ^13^.

En el presente estudio, no se identificaron otros factores de riesgo causantes de alteraciones neurológicas, enfermedades maternas gestacionales o ingestión de tóxicos o sustancias psicoactivas. La mayoría de los pacientes nacieron a término, con una edad gestacional media de 38 semanas; el 13 % de los pacientes nacieron prematuros, en su mayoría tardíos, y el peso y la talla media al nacer fueron adecuados para la edad gestacional.

Llama la atención que no se reportó retardo del crecimiento intrauterino, lo cual es una manifestación de la toxoplasmosis congénita, aunque en menos del 10 % de los casos ^15^. Al nacer, todos los pacientes tuvieron un adecuado índice de Apgar, sin datos de asfixia perinatal. El 15 % de los recién nacidos presentó enfermedades neonatales controladas, similar a lo reportado en otras series de seguimiento en América y Europa, en las cuales las tasas de embarazo pretérmino estaban alrededor del 10 %; la mayoría eran recién nacidos a término, con peso y talla adecuados para la edad, y sin datos de asfixia ^11^^,^^15^.

La edad promedio de diagnóstico posnatal de toxoplasmosis congénita fue de 9,4 meses: el 73 % de los pacientes fue diagnosticado antes del primer año y casi la mitad (46,6 %) de los diagnósticos se hicieron en el primer mes. En el presente estudio se encontraron diferencias estadísticamente significativas en la edad de diagnóstico de los pacientes con tratamiento posnatal (cerca de 22 meses), por lo cual es importante recalcar que el diagnóstico temprano es útil para iniciar una intervención a tiempo y evitar secuelas a largo plazo. Dicha diferencia de edad puede explicar por qué la tasa de tratamiento posnatal fue del 68 % y no del 100 %, ya que, como lo muestran los estudios, los pacientes sometidos a tratamiento fueron aquellos menores de un año ^21^.

La media de duración del tratamiento fue de 12 meses, como se indica en la guía nacional, mediante un esquema de pirimetamina, ácido folínico y sulfadiazina, o pirimetamina, ácido folínico y sulfadoxina cuando no se dispone de sulfadiazina; 6 (13 %) no recibieron ácido folínico, utilizado para prevenir efectos adversos asociados con el tratamiento. En los estudios sobre tratamiento, la sulfadiazina muestra la mayor evidencia clínica, aunque hay autores que respaldan el uso de sulfadoxina como agente equivalente ^3^^,^^8^.

Los pacientes sintomáticos al nacer en la cohorte aquí descrita correspondieron al 11 %, pero en los estudios colombianos previos esta tasa fue más alta: de 41 % en un estudio de 26 pacientes y de 95 % en otro de 21 casos. Sin embargo, esto puede explicarse por tratarse de cohortes de madres con infección gestacional confirmada en seguimiento, en estudios de hace dos décadas ^11^^,^^12^. En otras series de seguimiento en Estados Unidos, la tasa de pacientes sintomáticos al nacer fue similar, y osciló entre el 5 y el 15 % ^3^. En la serie descrita en este trabajo no hubo mortalidad neonatal por toxoplasmosis congénita, como sí se describió en el estudio multicéntrico colombiano de Gómez-Marín *et al.,* en el cual el 20 % de 15 pacientes con toxoplasmosis congénita falleció en el primer mes, y en otras dos series, el 11 % y el 5 %, respectivamente ^11^^,^^12^^,^^16^.

Es importante resaltar que el 33 % de los pacientes de esta cohorte tenía retinocoroiditis al nacer, un porcentaje cercano al informado en series colombianas previas, que fueron del 42 y el 34 % ^11^^,^^12^. Estas tasas son superiores a lo reportado en cohortes europeas, como la del estudio EMSCOT *(European Multicentre Study on Congenital Toxoplasmosis)* cercana al 10 %, y en otros países de Suramérica, como Brasil, donde la tasa de retinocoroiditis fue del 50 % en el primer año. Además, la tasa de defectos en la agudeza visual o secuelas oftalmológicas fue del 73 % en este seguimiento. En el presente estudio, fue similar a lo reportado en Brasil (87 %) y por encima de lo observado en Europa (29 %), lo que refuerza la teoría de que los genotipos del parásito que circulan en Suramérica podrían ser más virulentos ^22^^,^^23^.

En dos series estadounidenses, una de ellas el *National Collaborative Chicago-Based Congenital Toxoplasmosis Study* (NCCCT), en el cual el seguimiento se hizo entre 1981 y el 2004, se encontraron grandes tasas de secuelas, como calcificaciones intracraneales (85 %), coriorretinitis (85 %), hidrocefalia (50 %), retardo del desarrollo psicomotor (85 %), crisis epilépticas (81 %) y pérdida auditiva (14 %) a los cuatro años de edad. En otro estudio de seguimiento, entre 1991 y 2005, se encontraron tasas muy similares en pacientes sin tratamiento prenatal o posnatal, lo que demuestra el gran impacto de las secuelas de la infección, más frecuentes en ausencia de tratamiento gestacional o neonatal ^21^^,^^24^.

Los estudios europeos realizados en madres con tratamiento gestacional y posnatal, han mostrado tasas más bajas de compromiso clínico. En el estudio *Systematic Review on Congenital Toxoplasmosis* (SYROCOT) se reportaron tasas de lesiones oculares del 14 %, de calcificaciones del 9 % y de cualquier manifestación clínica del 19 % en el primer año de vida ^24^. Otros estudios de seguimiento a largo plazo, como el de Berrebi en Francia durante ocho años, mostraron tasas de sintomatología de solo del 28 % ^25^. Incluso, en el estudio SYROCOT se manejó una cohorte comparativa de Suramérica, específicamente de Colombia y Brasil. La serie colombiana incluyó ocho pacientes, con una tasa de manifestaciones clínicas entre 38 y 77 %, y específicamente oculares, del 47 %; en el examen neurológico, se hallaron lesiones intracraneales en el 53 %, comparativamente con el 13 % de la cohorte europea ^26^.

En esta serie colombiana se encontraron tasas de retinocoroiditis en el 75 % de la población, calcificaciones cerebrales en el 65 % e hidrocefalia en el 4,4 %, hipoacusia en el 6,6 %, parálisis cerebral en el 11,2 % y epilepsia focal en el 13 %. Además, se evaluó el compromiso del neurodesarrollo, que fue del 58 % en menores de seis años, y se determinó el déficit cognitivo en mayores de seis años, que afectó al 66 %. El compromiso oftalmológico parece ser el más importante, seguido del estado del neurodesarrollo y las habilidades cognitivas. Estos datos concuerdan con los de las cohortes suramericanas mencionadas anteriormente ^11^^,^^12^^,^^23^.

Para explicar las diferencias entre la gravedad de compromiso clínico entre las cohortes europeas y las americanas, se ha propuesto que los linajes circulantes de *Toxoplasma gondii* en el continente americano corresponden en su mayoría al I y al III, atípicos y más patógenos que los de tipo II circulantes en Europa ^27^.

Se ha demostrado que el tratamiento prenatal reduce la tasa de infección congénita y, a largo plazo, las secuelas neurológicas graves ^20^^,^^28^. Por su parte, el tratamiento posnatal (con sulfadiazina y pirimetamina) durante un año en pacientes con toxoplasmosis congénita, ha demostrado mejoría en los resultados neurológicos. En un estudio de seguimiento de 120 pacientes, se encontró que, en comparación con los controles históricos, el 72 % de los niños con compromiso neurológico mayor tenía puntuaciones cognitivas normales y ausencia de compromiso auditivo ^21^. Otros estudios han demostrado mejoría relacionada con la disminución de la prevalencia de la epilepsia, las alteraciones motoras y el tono muscular e, incluso, en la resolución de calcificaciones intracraneales, aunque con un número limitado de pacientes ^29^^,^^30^.

En la presente serie no se evidenció una asociación estadísticamente significativa entre el tratamiento prenatal o posnatal, y el riesgo de secuelas neurológicas y lesiones intracraneales, ajustado por trimestre de infección, como factor de confusión demostrado en otros estudios ^11^. Se encontró una asociación ajustada y estadísticamente significativa entre el riesgo de secuelas oftalmológicas y la ausencia de tratamiento posnatal, concordante con un estudio que muestra una tasa de lesiones oculares con tratamiento reducida al 30 %, lo que demuestra la importancia del tratamiento temprano ^31^. De igual manera, se observa que, con respecto a las cohortes no tratadas de los Estados Unidos, el riesgo de presentar secuelas neurológicas, lesiones intracraneales, epilepsia e hidrocefalia es bastante menor en el seguimiento realizado por los autores, lo que puede explicarse por el tratamiento prescrito; sin embargo, se requieren estudios con un mayor tamaño de muestra y enfocados en ese objetivo para poder demostrarlo.

El presente estudio puede presentar sesgo de selección, debido a que la población pediátrica más afectada es la que asiste al seguimiento, y sesgo de información, porque es un estudio basado en recolección de datos de historias clínicas.

En la evaluación del tratamiento prenatal o posnatal, se consideraron factores de confusión como el tiempo, la dosis y la adhesión al tratamiento, porque no se tienen datos relacionados. Por lo anterior, este estudio no debe tomarse para evaluar el tratamiento.

Como fortaleza, este es el primer estudio en el país que presenta el seguimiento a largo plazo de la toxoplasmosis congénita y sus secuelas clínicas y alteraciones del neurodesarrollo, y resalta la importancia de su diagnóstico y manejo tempranos.

En conclusión, se describe una cohorte de pacientes pediátricos con toxoplasmosis congénita, atendidos en el Hospital Militar Central entre el 2013 y el 2020, cuyas secuelas más importantes fueron las oftalmológicas (73 %). En la evaluación neurológica, el 58 % de los menores de seis años tenía compromiso del neurodesarrollo, y en los mayores de seis años, el 62 % presentó déficit cognitivo. Se encontró una asociación estadísticamente significativa entre no recibir tratamiento y la presencia de secuelas oftalmológicas en la cohorte presentada.

Este estudio sirve de base para reforzar la importancia de hacer un diagnóstico temprano, ofrecer un tratamiento oportuno y hacer seguimiento interdisciplinario a los pacientes con toxoplasmosis congénita en el país.
